# Pharmacokinetic-pharmacodynamic modelling to investigate *in vitro* synergy between colistin and fusidic acid against MDR *Acinetobacter baumannii*

**DOI:** 10.1093/jac/dky524

**Published:** 2019-01-08

**Authors:** Lynette M Phee, Frank Kloprogge, Rebecca Morris, John Barrett, David W Wareham, Joseph F Standing

**Affiliations:** 1 Centre for Immunobiology, Blizard Institute, Queen Mary University of London, London, UK; 2 Royal Free London NHS Foundation Trust, London, UK; 3 Great Ormond Street Institute of Child Health, University College London, London, UK; 4 UCL Institute for Global Health, University College London, London, UK; 5 Medicines Research Centre, GlaxoSmithKline, Stevenage, UK; 6 Barts Health NHS Trust, London, UK; 7 Great Ormond Street Hospital for Children NHS Trust, London, UK

## Abstract

**Objectives:**

The potential for synergy between colistin and fusidic acid in the treatment of MDR *Acinetobacter baumannii* has recently been shown*.* The aim of this study was to perform an extensive *in vitro* characterization of this effect using pharmacokinetic-pharmacodynamic modelling (PKPD) of time–kill experiments in order to estimate clinical efficacy.

**Methods:**

For six clinical strains, 312 individual time–kill experiments were performed including 113 unique pathogen–antimicrobial combinations. A wide range of concentrations (0.25–8192 mg/L for colistin and 1–8192 mg/L for fusidic acid) were explored, alone and in combination. PKPD modelling sought to quantify synergistic effects.

**Results:**

A PKPD model confirmed synergy in that colistin EC_50_ was found to decrease by 83% in the presence of fusidic acid, and fusidic acid maximum increase in killing rate (*E*_max_) also increased 58% in the presence of colistin. Simulations indicated, however, that at clinically achievable free concentrations, the combination may be bacteriostatic in colistin-susceptible strains, but growth inhibition probability was <20% in a colistin-resistant strain.

**Conclusions:**

Fusidic acid may be a useful agent to add to colistin in a multidrug combination for MDR *Acinetobacter baumannii.*

## Introduction

The rise of antimicrobial resistance has captured global attention with particular focus on MDR infections for which viable therapeutic options range from few to none.^1^ MDR *Acinetobacter baumannii* (MDRAB) is a prime example of a threat to modern medicine, with its predilection for the critically ill, affecting the success and progress of a wide range of medical fields including surgery and oncology.^2^

Colistin, first discovered in the 1940s and subsequently abandoned in clinical use for less toxic alternatives, is now increasingly used as an agent of last resort to combat MDR Gram-negative infections.^3^ In attempts to preserve the usefulness of colistin clinically, researchers have been investigating its use in combination with other licensed antimicrobial agents.^4^ This approach appears to present a number of advantages: relative increase in antimicrobial activity of the combination; potential reduction of the dose of colistin (thereby minimizing toxicity); retardation of the development of resistance; and enhancement of bactericidal activity and hence a cure for an infection that might not otherwise be curable with current monotherapies.^4^ Moreover, there have been reports of rapid evolution of colistin resistance during colistin monotherapy resulting in therapeutic failure.^4–6^ Colistin heteroresistance is a well-described phenomenon amongst *A. baumannii* isolates and has been thought to contribute to therapeutic failure with monotherapy, as heteroresistant isolates may appear to be susceptible to colistin *in vitro* by conventional susceptibility testing methods used in routine clinical laboratories (e.g. broth microtitre dilution). In an effort to investigate colistin susceptibility, as well as incorporate clinical dosing regimens to better predict clinical outcome, pharmacometric modelling on time-course experiments has been developed and explored in recent years.^7–9^

A systematic review by Zusman *et al*.^10^ has concluded that current evidence does not advocate for the use of previously described colistin combinations (e.g. colistin/rifampicin and colistin/tigecycline) despite synergy *in vitro* cited by many sources.^4^^,^^11^ More recently, Paul *et al*.^12^ concluded that there was a lack of evidence for the addition of meropenem to colistin for the treatment of carbapenem-resistant *A. baumannii* based on a large multicentre randomized controlled trial. This is in stark contrast to a meta-analysis of *in vitro* studies of polymyxin/carbapenem combinations by Zusman *et al*.^13^ citing strong synergistic antibacterial effect. This might be due, in part, to the overly simplistic way in which synergy is defined. Most commonly, chequerboard assays, which are based on determinations of MIC with or without exposure to the second agent,^14^^,^^15^ are used. Although synergy in chequerboards may indicate a useful mechanism, ensuring this effect persists at clinically achievable concentrations is crucial to predicting clinical utility.

Pharmacokinetic-pharmacodynamic (PKPD) modelling of *in vitro* experiments may better describe the interaction between two agents over time compared with chequerboard experiments, hence improving the prediction of their combined antimicrobial activity.^16–19^ Rao *et al*.^18^ described the lack of sustained synergy, which was observed initially between polymyxin B and tigecycline, when using current recommended doses. Modelling the time–kill curves provided possibilities for optimizing antibacterial activity by increasing doses of tigecycline and/or including a loading dose for polymyxin B.^18^

The aim of this study was to model time–kill experiments, to better understand the synergy we have previously observed between colistin and fusidic acid against *A. baumannii*^20^ using conventional methods of assessing synergy (i.e. chequerboards and time–kill experiments utilizing NCCLS definitions).^21^ In particular, the model seeks to address differences between antimicrobial activity of the combination against strains exhibiting high-level colistin resistance (MIC >256 mg/L) compared with heteroresistant strains. We then assessed the potential for *in vitro* synergy to translate into clinical dose recommendations using Monte Carlo simulation.

## Methods

### Bacterial strains

Six *A. baumannii* strains were selected to be included in the study: ATCC 19606 (antibiotic-susceptible type strain) and five MDR clinical isolates representing OXA-23 UK clone I (AB14 and AB315), OXA-23 UK clone II (AB16), SE clone (AB12) and a colistin-resistant strain (AB205) (isolates and typing information provided by J. Turton, Public Health England). Broth microtitre dilution colistin MICs were 0.25 mg/L for ATCC 19606; 0.5 mg/L for AB14 and AB16; 1 mg/L for AB12 and AB315; and >256 mg/L for AB205. Broth microtitre dilution fusidic acid MICs were 32 mg/L for AB205; 64 mg/L for AB12; 128 mg/L for AB14 and ATCC 19606; 256 mg/L for AB315; and 512 mg/L for AB16. All isolates were stored at −70°C and grown on unsupplemented Iso-Sensitest agar (ISA; Oxoid, Basingstoke, UK) under aerobic conditions at 37°C.

### Antibiotics

Stock solutions of colistin sulphate (Alfa Aesar, Haverhill, USA) and fusidic acid (Sigma–Aldrich, St Louis, USA) were prepared with sterile distilled water to yield concentrations of 10 000 mg/L and 50 000 mg/L respectively.

### Time–kill assays

Overnight broth cultures of each isolate were prepared in 10 mL of Iso-Sensitest broth (ISB; Oxoid). Time–kill assays were set up in 10 mL ISB with starting inocula of 10^6^ cfu/mL as per a previously published protocol.^20^ A separate experiment to investigate inoculum effect with AB14 was conducted using starting inocula ranging between 10^7^ and 10^8^ cfu/mL.

Concentrations of colistin used ranged from 0.25 to 8192 mg/L (colistin concentrations 1024–8192 mg/L were prepared directly in broth without the use of antibiotic stock solution) and fusidic acid concentrations from 1 to 8192 mg/L. Concentrations chosen ranged from suboptimal to maximal (or 8192 mg/L) bactericidal activity for each single- or dual-agent condition. Bacterial colony counts were determined by plating out 100 μL aliquots (with serial dilutions in sterile PBS where appropriate) onto unsupplemented Iso-Sensitest Agar (ISA), with manual counts performed following incubation under aerobic conditions at 37°C for 18–24 h.

### LC-MS/MS analysis

Simulated samples were prepared in parallel to investigate the changes to concentrations of colistin and fusidic acid with time, singly and in combination.

Samples and calibration standards were filtered using a Millex 0.22 μM low-binding fast-flow variety Polyethersulfone (PES) membrane filter unit. The filtrates were then extracted by protein precipitation [50 μL of sample + 250 μL of acetonitrile:water (70:30) containing 100 ng/mL of internal standard]. Samples were centrifuged at 13 000 rpm for 10 min in a microfuge. One hundred microlitres of supernatant was transferred into a 96-well plate for sample analysis.

Samples and calibration standards were analysed by LC-MS/MS. An Acquity BEH Protein C_4_ 1.7 μm 2.1 mm × 100 mm column was used for the analysis. Reversed-phase gradient chromatography was performed with 0.1% formic acid in 100% water (A) and 0.1% formic acid in 100% acetonitrile (B) at a flow rate of 0.4 mL/min. The gradient used was 0 min, 5% B; 4.50–4.70 min, 95% B; 4.80 min, 5% B; 5.30 min, stop. The injection volume was 5 μL.

Positive/negative ion-switching electrospray tandem mass spectrometry was performed using a Sciex API 6500 QTRAP mass spectrometer with multiple reaction monitoring (MRM) detection mode (controlled by Analyst 1.6.2 software). The ion spray voltage was set to 4000 V (±switching) and the probe temperature was set at 500°C. Nitrogen was used as the collision gas. The nebulizer gas (GS1), curtain gas and turbo gas (GS2) were set to 55, 40 and 55 psi, respectively. MRM parameters used are shown in Table 1. A 10 ms dwell time was used between each MRM.

**Table 1.** MRM parameters used for colistin A, colistin B and fusidic acid assays

Compound	MRM (m/z)	DP (V)	CE (V)	CXP (V)
Colistin A	585.4/241	156	29	12
Colistin B	578.7/227.2	151	29	22
Fusidic acid	515.3/221.0	−130	−36	−10

DP is the declustering potential, CE is the collision energy and CXP is the collision cell exit potential.

### Modelling

Concentration and cfu data transformed into their natural logarithm and logarithm base 10, respectively, were modelled using NONMEM 7.3 on a Windows 10 operating system. Minus twice the log likelihood of the data was used as objective function value (OFV). A drop in OFV of at least 3.84 points (*P = *0.05) after inclusion of one additional parameter (one degree of freedom) to a nested hierarchical model was considered to statistically improve the model’s ability to describe the data. Discrimination of hierarchical models was further supported by goodness-of-fit diagnostics and biological plausibility.

Colistin and fusidic acid concentration data were estimated using ADVAN9 and the FOCE-I estimation method. *A. baumannii* cfu data were estimated using ADVAN9 and the Laplacian estimation method in order to facilitate the use of the so-called ‘M3 method’ for including cfu counts below 10 cfu/mL.

Dynamic colistin and fusidic acid drug concentrations in broth over time were modelled to study drug degradation and/or binding within the time span of the *in vitro* static time–kill curve experiments (i.e. 24 h). Baseline concentrations were fixed to the observed value with an added residual error term of the same magnitude as the residual variability (Eq. 1).

(1) $$P_{i}=\theta_{BL}\times e^{\varepsilon }$$

*P*_i_ represents estimated individual baseline concentration by θ_BL,_ which was fixed to the observed baseline, with *ε* being the proportional residual error (additive on the log scale).

Drug degradation was described using a first-order process (Eq. 2).

(2) $$\frac{dC}{dt}=-\theta_{k}\times C$$

Changes in drug concentration (C) over time (t) in Eq. 2 were explained by a rate constant (θ_k_) and drug concentrations.

*A. baumannii* baseline cfu was evaluated using both observed and estimated (Eq. 1) initial conditions. Bacterial growth was described using a logistic model (Eq. 3) with lag time (Eq. 4).

(3) $$\frac{dcfu}{dt}=k\times cfu\times \left(1-\frac{cfu}{{\theta_{cfu}}_{max}}\right)$$

(4) $$k=-\theta_{net}+2\times \theta_{net}\left(\frac{t^{20}}{{\theta_{lag}}^{20}+t^{20}}\right)$$

The rate constant k (Eqs. 3 and 4) represents the net growth and θ_cfumax_ (Eq. 3) is the maximum carrying capacity. The underlying net growth in Eq. 4 is described by θ_net_ and the time where half of the maximum lag time occurs is described by θ_l__ag_.

The growth model was developed on antimicrobial-free data only. Growth parameters were subsequently fixed and used to estimate cfu data for AB14 strain *A. baumannii* exposed to a range of colistin or fusidic acid concentrations. Drug effects were described using a sigmoidal *E*_max_ model (Eq. 5) and a time-dependent drug effect component, representing the development of resistance, which was described using a Gompertz model.

(5) $$E=\frac{\theta_{E_{max}}\times C^{\theta_{\gamma }}}{\theta_{{EC}_{50}}^{\theta_{\gamma }}+C^{\theta_{\gamma }}}\;(1-\theta_{\beta }(1-e^{-t\left(\theta_{\tau }\times C\right)}))$$

C in Eq. 5 represents drug concentration; the drug effect (E) in Eq. 5 was described by the maximum drug effect (θ_*E*___max__), the concentration realizing half the maximum effect (θ_EC_50__) and a shape parameter (θ_γ_). The effect size of time-dependent drug effect was represented by θ_β_ (which was constrained to take values in the interval 0 to 1) in Eq. 5, the duration of the treatment effect was described by θ_τ_ and drug concentrations were represented by C. The basic model for change in cfu with time was therefore given by:

(6) $$\frac{dcfu}{dt}=(k-E)\times cfu\times \left(1-\frac{cfu}{{\theta_{cfu}}_{max}}\right)$$

The terms in E were further refined as follows: θ_β_ in the Gompertz time-dependent drug effect component was fixed for fusidic acid and drug combinations to the estimated value for colistin. Both static drug concentrations as well as dynamic drug concentrations, adjusted for drug degradation, were evaluated.

Bacterial burden (cfu) over time data from the ATCC 19606, AB12, AB16, AB315 and AB205 *A. baumannii* strains were sequentially included into the model. Altered drug susceptibility for each *A. baumannii* strain was assessed as categorical covariates (Eq. 7) with stepwise-covariate model building on *E*_max_, EC_50_, γ, β and τ.

(7) $$Covariate=(1+\theta_{cov})$$

The θ_cov_ in Eq. 7 represents the effect size of the categorical covariate.

*E*_max_ and time-dependent drug effect parameters (i.e. drug resistance development parameters) were fixed for colistin and fusidic acid and used to estimate cfu data for AB14 *A. baumannii* exposed to the colistin and fusidic acid drug combination at a range of concentrations. An additive colistin and fusidic acid drug effect was used as starting point. A categorical covariate (Eq. 7) on β for drug combinations was embedded in the model.

(8) $$Time–dependent\;effect=1-\theta_{\beta }(1-e^{-t((\theta_{\tau_{col}}\times C_{col})+(\theta_{\tau_{\mathrm{fus}}}\times C_{fus})+(\theta_{\tau_{comb}}\times C_{col}\times C_{fus}))})$$

The effect size of time-dependent drug effect was represented by θ_β_ in Eq. 8, the duration of the colistin (col), fusidic acid (fus), and combination (comb) treatment effect was described by θ_τ_ and corresponding drug concentrations were represented by C. This term was multiplied by the effect parameter, and hence effect decreases to 0 with increasing time and concentration.

Subsequently, colistin and fusidic acid interaction effects were assessed with stepwise-covariate model building using categorical covariate relations (Eq. 7) or an *E*_max_ model (Eq. 5), without *E*_max_ and γ estimated, on *E*_max_, EC_50_, γ, β and τ_c__omb_.

cfu-over-time data from the ATCC 19606, AB12, AB205, AB315 and AB16 *A. baumannii* strains were sequentially included into the model.

A non-parametric bootstrap (*n *=* *1000) was performed in order to derive the parameter standard errors. The prediction-corrected visual predictive check (VPC) enabled the assessment of the model’s predictive performance. The 5th, 50th and 95th percentiles of the observed data were overlaid with the 95% CIs of the simulated (*n *=* *2000) 5th, 50th and 95th percentiles of the data for drug concentrations.

### Simulations

Clinical PK models were identified for intravenous colistin (given as its prodrug, colistimethate sodium) and oral fusidic acid and used to simulate concentration–time profiles under standard and high dosing regimens.^22^^,^^23^ For intravenous colistimethate sodium therapy, standard dosing was set to 2 million units (MU) three times per day, and high dosing was a loading dose of 9 MU followed by 3 MU three times per day.^23^ For oral fusidic acid therapy, standard dosing was defined as 500 mg three times per day, with high dosing defined as a loading dose of 1500 mg followed by 750 mg three times per day. Simulations (*n *=* *1000) of free drug, assuming an unbound fraction of 64% and 10% for colistin and fusidic acid respectively, were applied to the final model, giving a predicted change in cfu/mL at 24 h. Comparison of the log_10_ decrease in cfu/mL under standard and high dosing was made for colistin or fusidic acid alone or the combination was made.

## Results

### Drug degradation experiments

Colistin and fusidic acid concentrations by LC-MS/MS were obtained for 103 simulated aliquots, ranging from 0 to 4 mg/L for colistin and 0 to 64 mg/L for fusidic acid, either singly or in combination, at timepoints 0, 2, 6 or 24 h.

**Figure 1.**
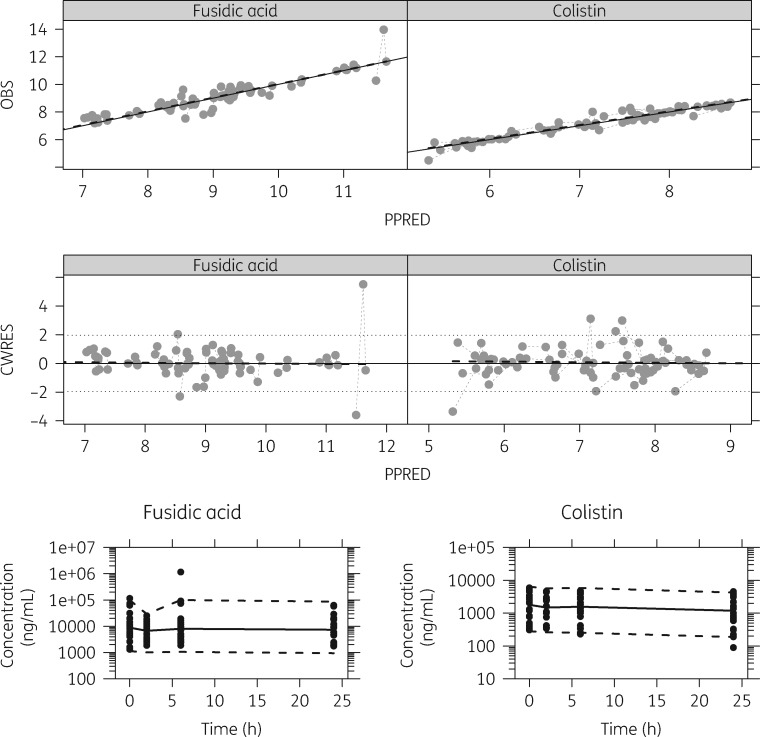
Basic goodness-of-fit and VPC plots for fusidic acid concentrations (left) and colistin concentrations (right). Top row: observed concentration (OBS) versus population prediction (PPRED) with line of unity and a dashed black line representing a smooth, and dashed grey line joining points from the same experiment. Second row: conditional weighted residuals (CWRES) versus PPRED, with and a dashed black line representing a smooth, a dashed grey line joining points from the same experiment and a black dotted line representing the expected 95% interval of the standard normal distribution. Bottom row: VPCs for fusidic acid concentrations (left) and colistin concentrations (right). The dashed and solid lines represent the 5th and 95th percentiles and the 50th percentile of the observed (black dots) concentration data, respectively.

Although fusidic acid concentrations remained constant, colistin displayed drug degradation and/or binding within the time span of the 24 h *in vitro* static time–kill curve experiments at a rate of 0.0175 h^−1^ [relative standard error (RSE): 20.9%] (Figure 1). In general, the model displayed good predictive performance for both colistin and fusidic acid (Figure 1).

Colistin drug degradation did not contribute to a significantly improved description of the *A. baumannii* cfu data (*P > *0.05) and was ultimately not included in the model.

### Time–kill experiments

Three hundred and twelve individual time–kill curves were included in the model, including 114 unique pathogen–antimicrobial combinations (27 colistin only, 33 fusidic acid only, 54 colistin/fusidic acid conditions) (see Supplementary Figures S1 to S4, available as Supplementary data at *JAC* Online). Eight high-inoculum experiments were performed in triplicate with no obvious inoculum effect on model fit (Figures S5 to S8). 

**Figure 2.**
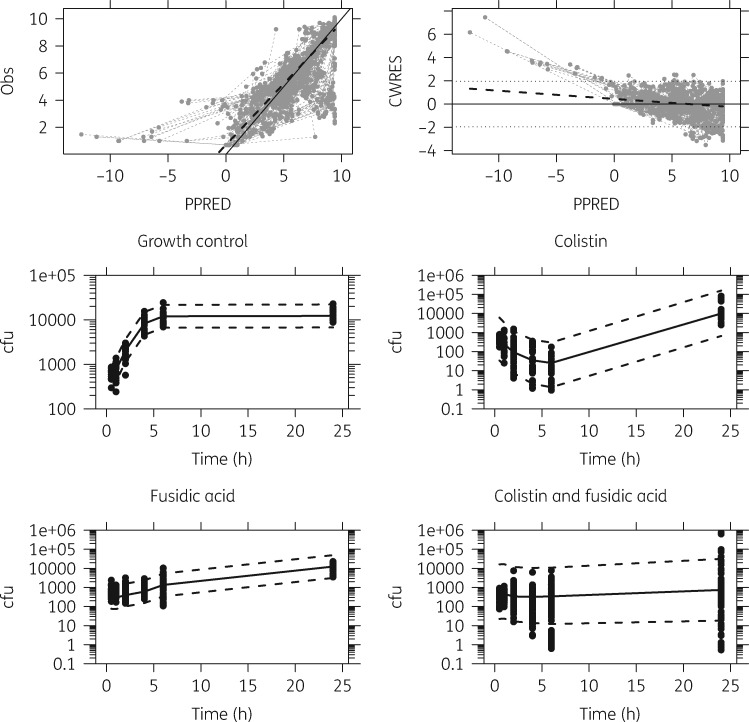
Top row: observed (Obs) versus population-predicted (PPRED) *Acinetobacter baumannii* cfu (left) and conditional weighted residuals (CWRES) versus PPRED (right) for the model with colistin and fusidic acid drug effect. The grey circles represent observed data, black dashed lines represent the locally estimated scatterplot smoothing (LOESS) trend lines, the black lines represent the lines of identity and dotted lines represent the 95% CI. Middle row: prediction-corrected VPC for *Acinetobacter baumannii* cfu growth (left) and colistin drug effect (right) model. Bottom row: prediction-corrected VPCs for *Acinetobacter baumannii* cfu fusidic acid drug effect (left) and colistin and fusidic acid drug effect (right) model. The black dots represent observations and the dashed and solid lines in the VPCs represent the mean 5th, 50th and 95th percentiles of the simulated (*n *=* *2000) data.

The *A. baumannii* cfu growth model fit was improved by the inclusion of a lag time (ΔOFV = −5.52; *P < *0.05) and was able to accurately and precisely describe the observed *A. baumannii* cfu growth data (Figure 2).

Both the colistin drug effect and fusidic acid drug effect were predicted well by the model (Figure 2). AB205 was substantially less susceptible to colistin (65.2-fold increase in EC_50col_) and more susceptible to fusidic acid (2.12-fold increase and −0.124-fold decrease in θ_τfus_ and θ_βfus_, respectively) (Table 2).

**Table 2.** Parameter estimates for the colistin and fusidic acid pharmacodynamics model

Parameter	Fixed effect (RSE)
θ_net_ (h^−1^)	1.87 (4.5)
θ_cfumax_ (cfu/mL)	9.41 (0.600)
θ_lag_ (h)	0.352 (14.4)
θ_EC50col_ (ng/mL)	9.86 (23.5)
proportional increase in θ_EC50col_ with AB205	65.2 (41.6)
θ_*E*__maxcol_ (h^−1^)	39.5 (10.6)
θ_γcol_	0.855 (4.60)
θ_τcol_	0.177 (38.6)
θ_βcol_	0.895 (1.10)
θ_EC50fus_ (ng/mL)	310 (43.9)
θ_*E*__maxfus_ (h^−1^)	23.2 (6.30)
θ_γfus_	0.776 (8.30)
θ_τfus_	0.0102 (37.2)
proportional increase in θ_τfus_ with AB205	2.12 (48.6)
θ_βfus_	0.895 (fixed)
proportional decrease in θ_βfus_ with AB205	−0.124 (22.3)
proportional decrease in θ_EC50col_ with fusidic acid	−0.826 (1.20)
proportional decrease in θ_EC50col_ with fusidic acid for AB205	−0.986 (0.200)
proportional increase in θ_*E*__maxfus_ with colistin	0.579 (52.2)
θ_τcomb_	1.44 (15.9)
θ_βcomb_	0.895 (fixed)
proportional decrease in θ_βcomb_ with AB12	−0.0523 (22.6)
Residual variability_growth model_	0.129 (15.9)
Residual variability_col_	2.8 (47.5)
Residual variability_fus_	0.698 (9.30)
Residual variability_comb_	5.14 (8.8)

**Figure 3.**
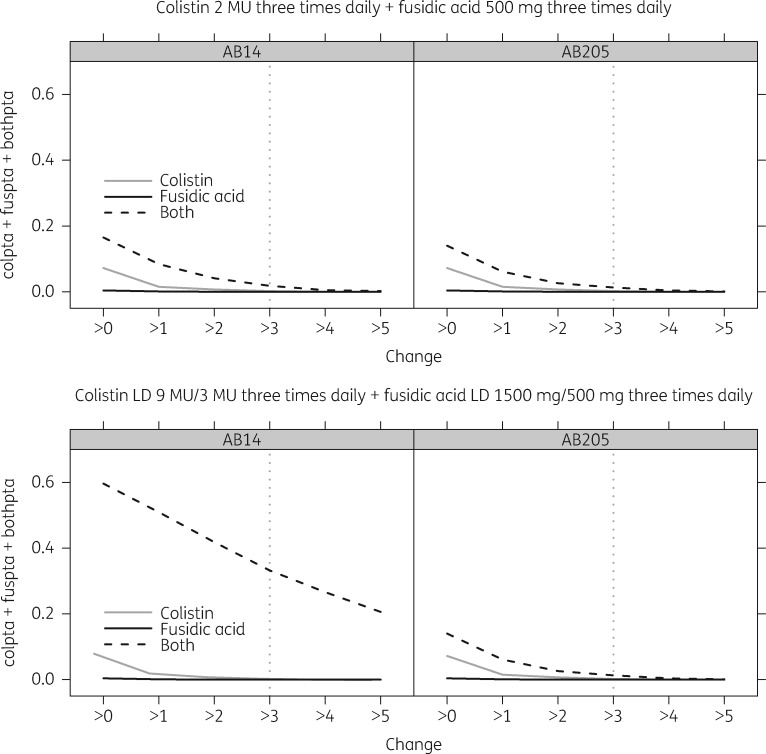
Probability of target attainment (PTA) versus the log_10_ cfu decrease in bacterial count at 24 h. Top row: standard dosing regimen with colistin and fusidic acid. Bottom row: optimized dosing regimen with colistin and fusidic acid. AB14 represents the most common and drug-susceptible *Acinetobacter baumannii* strain and AB205 represents a colistin-resistant *Acinetobacter baumannii* strain. LD, loading dose.

In general, the model provided a good description of the cfu data, and simulated trends match those observed (Figure 2). Moreover, the individual prediction versus observed cfu plots and individual model-predicted trajectories over time, stratified by treatment combination, indicate that the majority of the data are reasonably well described. There was substantial interaction between colistin and fusidic acid, with fusidic acid reducing EC_50col_ by 82.6% and colistin increasing *E*_max__fus_ by 57.9% (Table 2). AB12 displayed a decreased colistin/fusidic acid combination resistance (Table 2). Simulations of probability of the degree of 24 h cfu decrease are given in Figure 3.

## Discussion

MDR Gram-negative infections, for which therapeutic options are rapidly dwindling, are a current problem requiring urgent attention.^24^^,^^25^ One of the most successful Gram-negative organisms to date is *A. baumannii*; its success is due to its ability to acquire and express phenotypic resistance to multiple classes of antibiotics, its resistance to numerous biocides and persistence on fomites, and its ability to cause a diverse range of infections, particularly in the critically ill population.^26–28^ Unfortunately, MDRAB has become a common infection globally, to which only agents of last resort (e.g. polymyxins and tigecycline), if any, may retain activity. Fear of therapeutic failures on polymyxin monotherapy has led to clinicians employing a range of combination therapies. Colistin, which is the predominantly used polymyxin, has seen a renaissance, with numerous reports in literature of its use with a range of other antimicrobial agents, often broad spectrum, with varying results.^11^ Our major finding is that although the addition of fusidic acid improves the bacterial killing rate, the double combination is insufficient for sustained bacteriostatic activity at clinically achievable concentrations.

The prevailing methods of investigating synergy between antimicrobial combinations do not sufficiently describe the antibacterial effect, and this may underlie the discrepancies observed between *in vitro* observations and clinical efficacy.^29^^,^^30^ Methodologies, such as chequerboard assays, although attractive for screening large numbers of combinations, may be inferior to time-course methods, as analysis of drug interaction and the consequent antibacterial effect is based on a single endpoint result. Moreover, these results do not provide any information regarding bactericidal activity, which is an important factor for accurate prediction of clinical efficacy.^20^^,^^31^

Dynamic models are extremely labour intensive^32^ and thus limit the number of different conditions (i.e. concentrations of each agent, either singly or in combination) one can input into the model, decreasing the accuracy of the model. Static time–kill assays were chosen [with modelling of concentrations of both drugs singly and in combination via an LC-MS/MS method to take into account the natural attrition of the drug(s) over 24 h] as a compromise to assess the novel antibiotic combination, colistin/fusidic acid. We have previously published data on the synergy observed against *A. baumannii* (including MDR and XDR strains) using chequerboard assays and time–kill assays employing the NCCLS definition (i.e. ≥2 log_10_ cfu/mL decrease in bacterial colony count at 24 h in the combination arm compared with either single agent), as well as the relative reduction in mutational resistance *in vitro* in the combination arm compared with single-agent exposure.^20^ Here, we utilized a modelling approach to describe the synergistic effect of the colistin/fusidic acid combination and, importantly, using simulation to give a probability of success (Figure 3).

We conducted most experiments on AB14 (typical clinical strain in the UK, OXA-23 UK clone I), and therefore colistin, fusidic acid and the colistin/fusidic acid drug combination effect models were initially developed using this strain. The drug effect model was developed by sequentially including colistin, fusidic acid and colistin/fusidic acid time–kill data. Simultaneous estimation of all model parameter estimates with the final model gave convergence problems and hence parameters were fixed in a stepwise fashion from a drug-free model, to individual colistin and fusidic acid effect, through to the combination model. This meant that model misspecification arising from our simple model could not bias parameter estimates of drug-free and single drug experiments when adding the combination data, although it may inflate unexplained variability. This approach yielded a good fit to the data (Figure 2) and a recent study highlighting mathematical identifiability problems of more complex modelling approaches, particularly in the context of limited experimental designs, also supports our somewhat empirical modelling approach.^33^ A limitation of the modelling work presented here is the empirical nature of the time- and concentration-dependent decrease in drug effect and hence we did not try to extrapolate beyond 24 h with our simulations, to answer questions such as optimal duration of therapy.

The drug combination effects have been parameterized as categorical covariates and resistance development in the cultures was described using an empirical Gompertz equation. More complex parameterizations to describe resistance development^34^ and drug combination effects^35^ were tested although this did not result in statistically significant improved and/or robust models. This was again in line with Jacobs *et al*.^33^ who showed that parameters in mechanistic models developed using total cfu data from time–kill experiments can be unidentifiable.

The model used has quantified the synergy observed between colistin and fusidic acid against MDRAB (fusidic acid reduced EC_50__col_ by 82.6%). Moreover, the model has quantified the decreased colistin effect in the colistin-resistant strain, AB205 (i.e. EC_50__col_ was 65.2-fold higher), although fusidic acid was more potent (i.e. 2.12-fold increase and −0.124-fold decrease in τ_fus_ and β_fus_, respectively) (Table 2). AB12 displayed a decreased colistin/fusidic acid combination resistance effect (Table 2).

Having shown synergy with our model, the simulation results (Figure 3) highlight the caution required in translating *in vitro* findings to the clinic. Whilst the addition of fusidic acid to a colistin regimen clearly does improve the probability of bactericidal activity (>3 log_10_ cfu/mL decrease), the high protein binding of fusidic acid does appear to limit this somewhat. A possible explanation might be that the observed serum concentrations do not wholly predict intracellular concentrations of fusidic acid. Lemaire *et al*.^36^ found that intracellular concentrations of fusidic acid (in macrophages) reached five times that of extracellular compartments and may explain the greater *in vivo* activity of fusidic acid compared with the observed serum concentrations. Additionally, it has been proposed that gastrointestinal reflux plays a major role in the development of ventilator-associated pneumonia (VAP), with the acidic environment causing mucosal damage and neutralization of pulmonary macrophages.^37^ This acidic environment, however, may further drive the intracellular accumulation of fusidic acid,^36^ resulting in disproportionately greater antimicrobial effect than predicted by the model. Finally, *in vitro* approaches are unable to fully incorporate the effect of host immune factors in response to infection.^38^ It remains possible that a sufficient reduction in bacterial load, without the consequent replacement of the initial susceptible population with a resistant or heteroresistant subpopulation,^39^ even without evident bactericidal activity as defined by guidelines, may result in a positive outcome with the cooperative intervention of host immune factors. However, based on our results it would seem that a triple combination with a yet-to-be-defined third agent will be required to be sure of clinical effect.

### Conclusions

The colistin/fusidic acid combination presents a novel approach for the treatment of *A. baumannii* infection. Utilizing PKPD modelling, we have illustrated the potency of this combination, and our findings do appear to show that some benefit would be derived from adding fusidic acid to a colistin regimen in a patient infected with *A. baumannii.* A dosing regimen of 9 MU followed by 3 MU three times per day for intravenous colistimethate sodium, and a loading dose of 1500 mg followed by 750 mg three times per day for oral fusidic acid, will maximize the potential benefit.

## Supplementary Material

Supplementary Data
