# Supplementary material for: Pharmacokinetic-pharmacodynamic modelling to investigate in vitro synergy between colistin and fusidic acid against MDR Acinetobacter baumannii
Source: J Antimicrob Chemother. 2019 Jan 8;74(4):961–9. doi: 10.1093/jac/dky524 (PMC6419616; doi:10.1093/jac/dky524)
Supplement: Supplementary Data [file dky524_supplementary_data.docx]

Supplementary data

Pharmacokinetic-pharmacodynamic modelling to investigate in vitro synergy between colistin and fusidic acid against multi-drug resistant Acinetobacter baumannii.

Table of Contents

[Growth curve data plots 2](#_Toc527379219)

[Colistin drug effect data plots 3](#_Toc527379220)

[Fusidic-acid drug effect data plots 4](#_Toc527379221)

[Drug combination data plots 5](#_Toc527379222)

[Inoculum stratified growth curve data VPC 6](#_Toc527379223)

[Inoculum stratified colistin drug effect data VPC 7](#_Toc527379224)

[Inoculum stratified fusidic-acid drug effect data VPC 8](#_Toc527379225)

[Inoculum stratified colistin/fusidic-acid drug effect data VPC 9](#_Toc527379226)

# Growth curve data plots


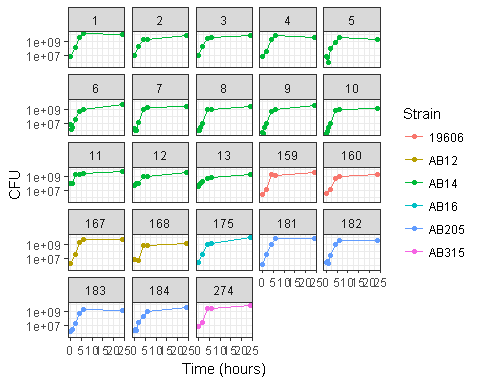


Figure S1: Individual CFU-time curve data plots for growth control experiments.

# Colistin drug effect data plots


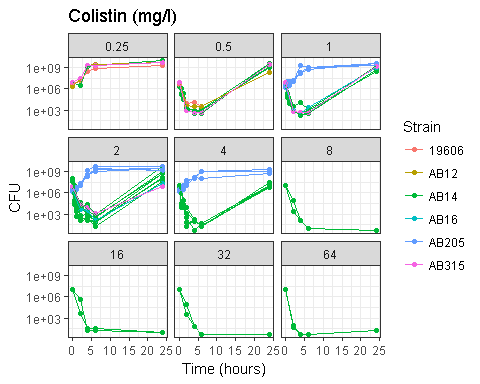


Figure S2: Individual CFU-time curve data plots for colistin experiments.

# Fusidic-acid drug effect data plots


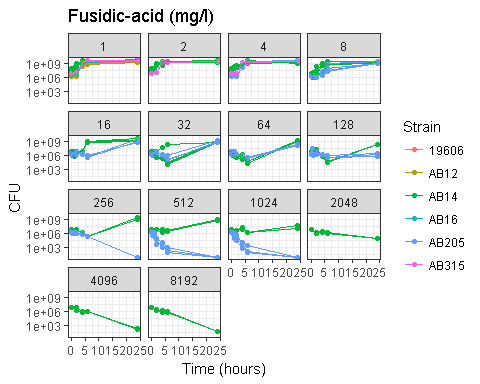


Figure S3: Individual CFU-time curve data plots for fusidic-acid experiments.

# Drug combination data plots


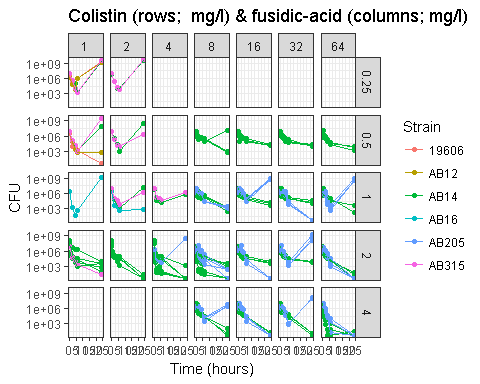


Figure S4: Individual CFU-time curve data plots for colistin and fusidic-acid combination experiments.

# Inoculum stratified growth curve data VPC


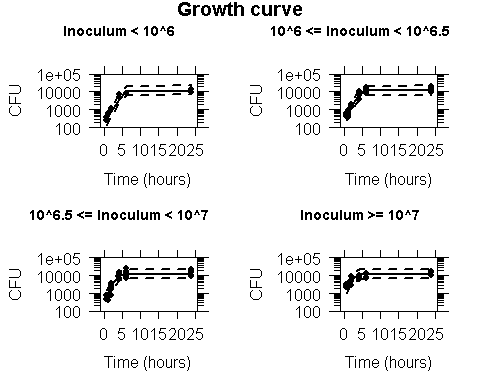


Figure S5: Visual Predictive Check for growth control experiment, stratified by inoculum.

# Inoculum stratified colistin drug effect data VPC


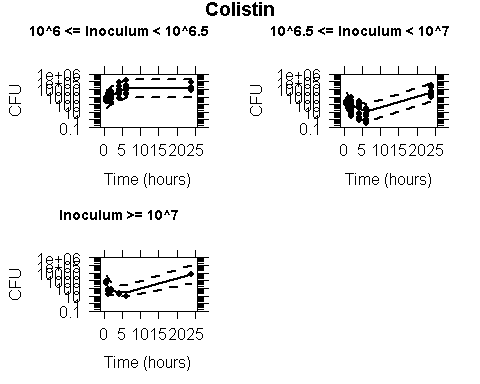


Figure S6: Visual Predictive Check for colistin experiments, stratified by inoculum.

# Inoculum stratified fusidic-acid drug effect data VPC


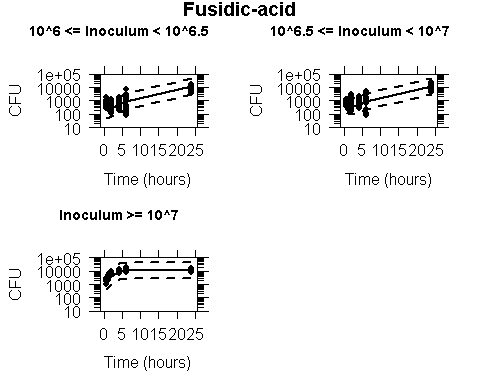


Figure S7: Visual Predictive Check for fusidic-acid experiments, stratified by inoculum.

# Inoculum stratified colistin/fusidic-acid drug effect data VPC


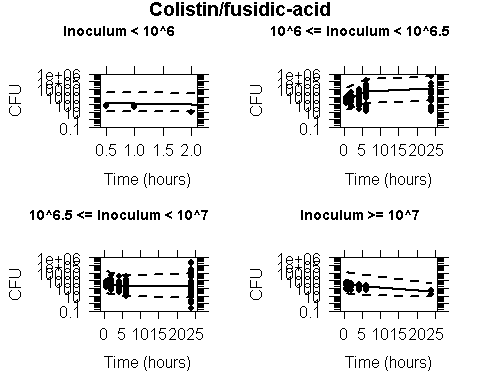


Figure S8: Visual Predictive Check for colistin and fusidic-acid combination experiments, stratified by inoculum.
